# Brusatol-Enriched Brucea javanica Oil Ameliorated Dextran Sulfate Sodium-Induced Colitis in Mice: Involvement of NF-*κ*B and RhoA/ROCK Signaling Pathways

**DOI:** 10.1155/2021/5561221

**Published:** 2021-08-09

**Authors:** Xinghan Zheng, Liting Mai, Tongtong Wang, Ying Xu, Zireng Su, Jiannan Chen, Huifang Zeng, Youliang Xie

**Affiliations:** ^1^School of Pharmaceutical Sciences, Guangzhou University of Chinese Medicine, Guangzhou 510000, China; ^2^The First Affiliated Hospital of Chinese Medicine, Guangzhou University of Chinese Medicine, Guangzhou 510000, China; ^3^Shandong Qingdao No. 2 Health School, Qingdao 266300, China

## Abstract

Brucea javanica oil (BJO) is beneficial for the treatment of ulcerative colitis (UC), and that quassinoids in particular brusatol are bioactive components. However, it is still uncertain whether or not other components in BJO, such as oleic acid and fatty acids, have an anti-UC effect. The present study is aimed at comparing the anti-UC effects between brusatol-enriched BJO (BE-BJO) and brusatol-free BJO (BF-BJO) and at exploring the effects and mechanisms of BE-BJO on colon inflammation and intestinal epithelial barrier function. Balb/C mice received 3% (wt/vol) DSS for one week to establish the UC model. Different doses of BE-BJO, BF-BJO, or BJO were treated. The result illustrated that BE-BJO alleviated DSS-induced loss of body weight, an increase of disease activity index (DAI), and a shortening of colon, whereas BF-BJO did not have these protective effects. BE-BJO treatment improved the morphology of colon tissue, inhibited the production and release of TNF-*α*, IFN-*γ*, IL-6, and IL-1*β* in the colon tissue, and reversed the decreased expressions of ZO-1, occludin, claudin-1, and E-cadherin induced by DSS but augmented claudin-2 expression. Mechanistically, BE-BJO repressed phosphorylation of NF-*κ*B subunit p65, suppressed RhoA activation, downregulated ROCK, and prevented phosphorylation of myosin light chain (MLC) in DSS-treated mice, indicating that the protective effect of BE-BJO is attributed to suppression of NF-*κ*B and RhoA/ROCK signaling pathways. These findings confirm that brusatol is an active component from BJO in the treatment of UC.

## 1. Introduction

Ulcerative colitis (UC) is a chronic bowel disorder characterized by diffuse inflammation of the mucosa of the colon and rectum [1, 2]. The hallmark clinical symptoms of UC include abdominal pain, rectal bleeding, and diarrhea [3]. Persistent UC leads to the development of colorectal cancer [4]. Currently, the etiopathogenesis of UC has not been completely understood. Although several categories of therapeutic drugs, such as anti-inflammatory drugs and immunosuppressants, have improved the life expectancy of patients, available effective therapies are still limited and the resurgence of diseases often occurs [5–7]. Therefore, development of effective therapeutic drugs for the management of UC would have important clinical implications.

*Brucea javanica* (L.) Merr. (Ya-dan-zi in Chinese, Simaroubaceae) is a crucial Chinese folk medicine widely used for the treatment of dysentery, which was also known as inflammatory bowel disease (IBD) [8, 9]. *Brucea javanica* oil (BJO), an extract of the desiccative ripe fruit of the *Brucea javanica* (L.) Merr., exhibits various bioactivities, including anti-inflammatory, antimalarial, and antitumor activities [10]. Our previous studies have shown that BJO protects against IBD in both 2,4,6-trinitrobenzenesulfonic acid- (TNBS-) induced Crohn's disease model and dextran sulfate sodium- (DSS-) induced UC model, via suppression of NF-*κ*B activation [11, 12]. Chemically, BJO is composed of flavonoids, alkaloids, volatile oils, sterols, fatty acids, and polyphenolic acids. Among all these components, quassinoids are vital bioactive compounds that display various types of biological activity [13, 14]. Indeed, we have reported that quassinoids, in particular, brusatol and bruceine D, had potential therapeutic effects against UC [15, 16]. However, it is still uncertain whether or not other components such as oleic acid and fatty acids have an anti-UC effect. Thus, we prepared BJO enriched with brusatol and brusatol-free BJO which mainly consists of oleic acid and fatty acids [17]. The present study is aimed at comparing the anti-UC effects between brusatol-enriched BJO (BE-BJO) and brusatol-free BJO (BF-BJO).

Moreover, inflammation and epithelial barrier defects in the alimentary system can damage the normal function of colon and promote the pathogenesis of UC. Since the anti-inflammatory effects of BJO and brusatol have been proven, the present study focused on investigating the potential effect of BJO on improving intestinal epithelial barrier function and repairing mucosal epithelium. This study will also try to elucidate the possible mechanisms of BJO, by investigating the involvement of the RhoA/Rho-associated serine-threonine protein kinase (ROCK) signaling pathway, which is pivotal in the process of intestinal inflammation and epithelial barrier dysfunction [18].

## 2. Materials and Methods

### 2.1. Materials

*Brucea javanica* oil emulsion was provided by Ming Xing Pharmaceutical Co. Ltd. (Guangzhou, Guangdong, China). Mesalazine (5-aminosalicylic acid, 5-ASA) was purchased from Germany Losan Pharma GmbH Co., Ltd. DSS (molecular weight: 36000~50000) was bought from MP Biomedicals (Canada). Brusatol (HPLC purity > 98%) was provided by the Guangdong Provincial Key Laboratory of New Drug Development and Research of Chinese Medicine. The preparation of BE-BJO and BF-BJO was based on our previous study [17]. Briefly, BJO was macerated with 95% ethanol (1 : 5 weight/volume) and extracted by ultrasonic solvent extraction in an ultrasonic bath at room temperature (40 kHz, 250 W) for 3 times to combine the supernatant. The supernatant was called brusatol-enriched BJO (BE-BJO), and the lower liquid was called brusatol-free BJO (BF-BJO). Primary antibodies (ZO-1, MLC, PMLC, occludin, claudin-1, p65, p-p65, RhoA, p-RhoA, E-cadherin, and ROCK-1) were purchased from Affinity Biosciences (CA, USA). *β*-Actin was purchased from Abcam (Shanghai, China). The enzyme-linked immunosorbent assay (ELISA) kits for TNF-*α*, IL-6, IL-1*β*, IFN-*γ*, and IL-10 were obtained from eBioscience (MA, USA).

### 2.2. HPLC Analysis

The HPLC analysis of brusatol in BE-BJO and BF-BJO was performed according to our previous investigation [17]. Measurements were carried out using HPLC LC-20AT (Shimadzu, Kyoto, Japan). PRONTOSIL120-3-C18-ace-EPS column (2.0 mm × 50 mm, 3.0 *μ*m, Bischoff, Germany). The injection volume was 10 *μ*L, the detection wavelength was 254 nm, and the he flow rate was set at 0.5 mL/min.

### 2.3. Animals

Healthy male Balb/C mice, weighing 22-25 g, were purchased from the Laboratory Animal Center of Guangzhou University of Chinese Medicine (Guangzhou, China). The animals were housed under standard environmental conditions where the temperature (23 ± 1°C), lighting condition (12 h light/dark cycles), and humidity (40–60%) are tightly controlled. The animals had free access to standard diet and water ad libitum. Animal procedures used in this study were in line with institutional guidelines for the Care and Use of Laboratory Animals (NIH Publication No. 85-23, revised 1996).

### 2.4. Induction of Colitis and Drug Administration

Acute colitis was induced by 3% (wt/vol) DSS dissolved in drinking water given ad libitum in Balb/C mice for 7 days. All mice were randomly assigned into seven groups: (1) normal group, received drinking water without DSS; (2) DSS group, given drinking water with 3% DSS and received soybean lecithin suspension throughout the experimental period; (3) 5-ASA group and BJO group, receiving 3% DSS and administrated with 30 mg/kg 5-ASA and 2000 mg/kg BJOE, respectively; (4) BE-BJO groups, receiving 3% DSS and administrated with BE-BJO 152.5 mg/kg/day (low dose, BE-BJOL), 305 mg/kg/day (medium dose, BE-BJOM), or 610 mg/kg/day (high dose, BE-BJOH), respectively, for 7 days.

Body weight was measured daily by using a digital weight scale. After 7 days, all mice were sacrificed after being fasted for 12 h and anesthetized by carbon dioxide (CO_2_) inhalation; then, the blood was collected immediately. Colon tissues were quickly removed, and its length was measured. Then, the colon tissues were collected for morphological observation, Western Blot, ELISA, and quantitative real-time polymerase chain reaction (qRT-PCR).

### 2.5. Evaluation of Disease Activity Index (DAI)

The index of disease activity was evaluated according to a previously reported procedure [12]. Briefly, DAI was scored as follows: DAI = (score of body weight loss + score of stool properties + score of hematochezia)/3. The criteria for evaluation are listed in Table 1.

### 2.6. Cytokine Analysis by ELISA

The levels of TNF-*α*, IL-6, IL-1*β*, IFN-*γ*, and IL-10 in colon tissues were measured quantitatively by using ELISA kits in accordance with the manufacturer's protocol. The levels of cytokine were quantified by standard curves.

### 2.7. Histological Analysis

The colon tissues were immediately fixed with 10% buffered formalin following harvest, then embedded in paraffin, and cut into 5 *μ*m thick sections. Finally, sections were stained with hematoxylin and eosin (H&E) for histopathological examination. A macroscopic score was evaluated according to the established histological grade [19] and is shown in Table 2.

### 2.8. Western Blotting Analysis

The colon segments from each group were homogenized and lysed using cold Pro-prep protein lysis buffer (Intron Biotechnology, Seoul, Republic of Korea) with addition of protease inhibitor cocktail and phosphatase inhibitors, for 30 min on ice. The tissue lysates were centrifuged, and the supernatant was collected. The BCA assay kit (Thermo Scientific Pierce, IL, USA) was used to determine the protein concentrations of the supernatant. Equal amounts of protein extract for each sample were separated in 8% or 10% of sodium dodecyl sulfate- (SDS-) polyacrylamide gel and transferred to a polyvinylidene difluoride (PVDF) membrane. The PVDF membranes were blocked with 5% skimmed milk for 1 h at room temperature. After that, the membranes were incubated overnight at 4°C with primary antibodies (dilution, 1 : 1000) against ZO-1, MLC, PMLC, claudin-1, claudin-2, p65, p-p65, RhoA, p-RhoA, E-cadherin, ROCK-1, and *β*-actin. The membranes were washed with TBS-T, followed by incubation with appropriate horseradish peroxidase- (HPR-) conjugated secondary antibody (dilution, 1 : 5000) for 1 h at room temperature. Next, the membranes were rinsed 3 times with TBS-T buffer and the signals of protein level were visualized using a Western imaging system. The intensity of the protein bands was quantified using Image J software. *β*-Actin was used as an internal control for total proteins.

### 2.9. Quantitative Real-Time Polymerase Chain Reaction (qRT-PCR)

qRT-PCR was implemented as described previously [20]. Briefly, the colon samples were homogenized with Trizol reagent. And then, total RNA was extracted according to the manufacturer's instructions. Quantitated RNA was reverse-transcribed into cDNA, and quantitative real-time PCR amplification was performed using the SYBR Green reagent to examine the mRNA expressions of ZO-1, occludin, and claudin-1. The primer sequences are listed in Table 3.

### 2.10. Statistical Analysis

The results were analyzed by using GraphPad Prism software 8.0 (GraphPad Software, La Jolla, CA, USA) and presented as mean ± standard error of mean (SEM). The numbers of mice in each group were shown as “*n*” number. Statistical significance was identified by using the one-way analysis of variance (ANOVA). *P* < 0.05 was considered to be statistically significantly different.

## 3. Results

### 3.1. BE-BJO Alleviated DSS-Induced Colitis in Mice

**Figure 1:**
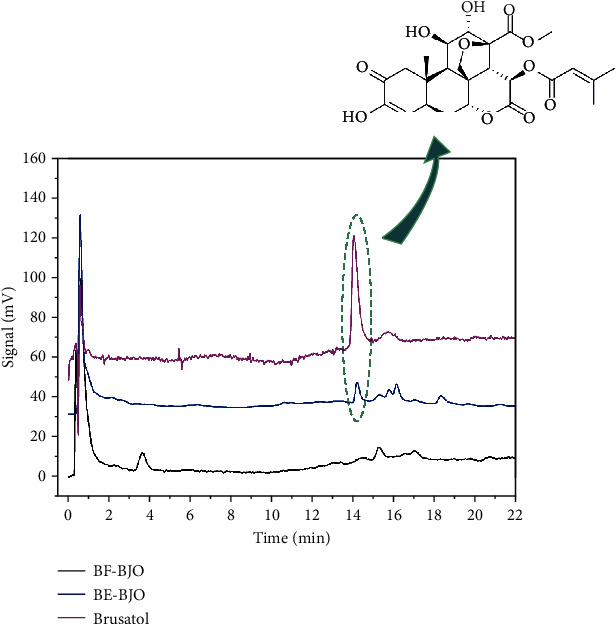
Comparative analysis of brusatol, BE-BJO, and BF-BJO using HPLC. The peak of standard compound of brusatol was detected at 14.5 minutes. The peak of brusatol was obviously detected in BE-BJO, but not in BF-BJO.

**Figure 2:**
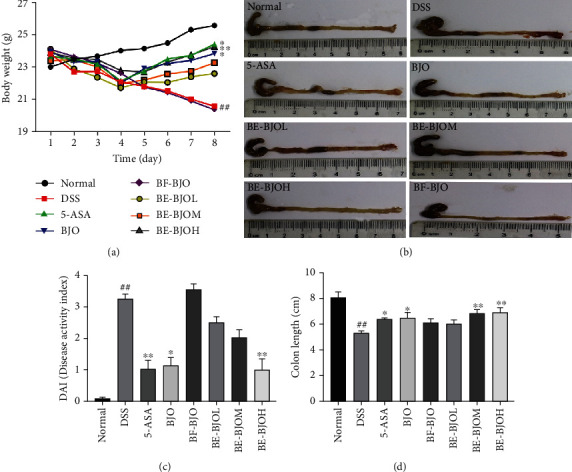
BE-BJO attenuated the severity of DSS-induced colitis in mice. Mice were monitored daily in terms of reductions of body weight (a), DAI score (b) in the DSS-induced UC mouse model. Representative photographs of colon length (c) and column chart of colon length (d). All values are presented as the mean ± SEM. ^##^*P* < 0.01 versus normal group; ^∗^*P* < 0.05, ^∗∗^*P* < 0.01, and ^∗∗∗^*P* < 0.001 versus DSS group.

Firstly, the existence of brusatol was examined in BE-BJO and BF-BJO. As demonstrated in Figure 1, brusatol was detected in BE-BJO, but not BF-BJO. To investigate the potential therapeutic effect of BE-BJO and BF-BJO on UC, the mice were treated with 3% DSS for 7 days to induce colitis. The body weights of the mice were tested; DAI including body weight loss, stool properties, and hematochezia were evaluated; and the length of the colon was measured. As shown in Figure 2, the DSS model group demonstrated a significant decrease in body weight, an increase in DAI scores, and a shortening of colon length. Treatment with BE-BJO alleviated DSS-induced colitis in a dose-dependent manner, as implied by the rebound of body weight and colon length, and the improvement of DAI (Figure 2). The effects of BE-BJO were similar as that of the positive control drug 5-ASA and BJO (Figure 2). By contrast, treatment with BF-BJO did not display protective effects against DSS-induced colitis (Figure 2). Thus, these observations suggest that BE-BJO, but not BF-BJO, is the active constituent from BJO that alleviates DSS-induced colitis in mice.

### 3.2. BE-BJO Prevented Histopathological Change in the Colon of DSS-Treated Mice

**Figure 3:**
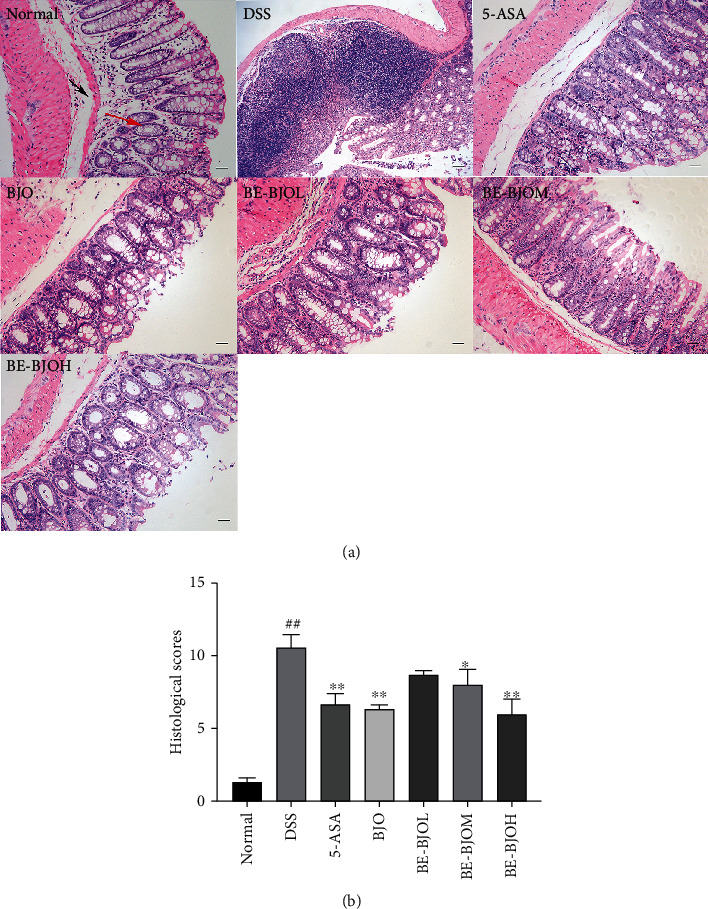
BE-BJO relieved the colonic injury in DSS-induced UC mice. The representative images of H&E staining of mice in each treatment group (magnification, 200x) (a) and histopathological score (b). The red arrow indicates the mucous layer, and the black arrow indicates submucosa. All values are presented as the mean ± SEM. ^##^*P* < 0.01 versus normal group; ^∗^*P* < 0.05 and ^∗∗∗^*P* < 0.001 versus DSS group.

The colon tissues were examined microscopically to observe the morphological changes of structure and integrity. The histopathologic features and scores were evaluated. DSS induced a destruction of the colon architecture, which was exhibited by obvious necrosis, epithelial damage or shedding, and submucosal infiltration of inflammatory cells, accompanied by crypt hyperplasia (Figure 3(a)). Treatment of BJO, 5-ASA, and BE-BJO (305, 610 mg/kg/day), but not BF-BJO, significantly improved the collapse of the colon structure and infiltration of inflammatory cells (Figure 3(a)). As shown in Figure 3(b), the histopathological score of the DSS group was significantly higher than that of the normal group; treatment with BJO and 5-ASA significantly reduced the histopathological score as compared to the DSS group; treatment of BE-BJO (305 and 610 mg/kg/day) significantly reversed DSS-induced increase of histopathology score, with a similar trend as BJO and 5-ASA. These results confirm that BE-BJO prevents colon histopathological change induced by DSS.

### 3.3. BE-BJO Attenuated the Levels of Proinflammatory Cytokines in the Colon of DSS-Treated Mice

**Figure 4:**
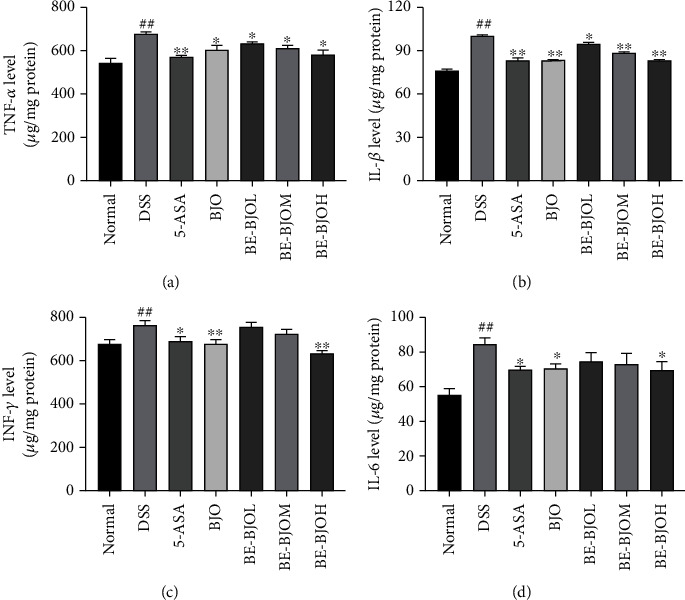
BE-BJO inhibited DSS-triggered inflammation. The expression of proinflammatory cytokines TNF-*α* (a), IL-1*β* (b), IFN-*γ* (c), and IL-6 (d) was detected by ELISA in colon sections. All values are presented as the mean ± SEM. ^##^*P* < 0.01 versus normal group; ^∗^*P* < 0.05, ^∗∗^*P* < 0.01, and ^∗∗∗^*P* < 0.001 versus DSS group.

Colon inflammation plays a key role in the development of UC [21–24]. The effects of BE-BJO on the production of proinflammatory cytokines, including TNF-*α*, IL-1*β*, IFN-*γ*, and IL-6, were examined in the colon tissue of DSS-treated mice. As displayed in Figure 4, the levels of these proinflammatory cytokines were significantly upregulated in colorectums of the DSS group; treatment of BE-BJO dose-dependently reversed DSS-induced production of proinflammatory cytokines. In addition, the anti-inflammatory effect of BE-BJO at a high dose (610 mg/kg/day) was equivalent to that of BJO and 5-ASA (Figure 4). Taken together, these data indicate that BE-BJO could ameliorate colon inflammation in the DSS model.

### 3.4. BE-BJO Restored the Impairment of Intestinal Epithelial Barrier Function in DSS-Induced Colitis

**Figure 5:**
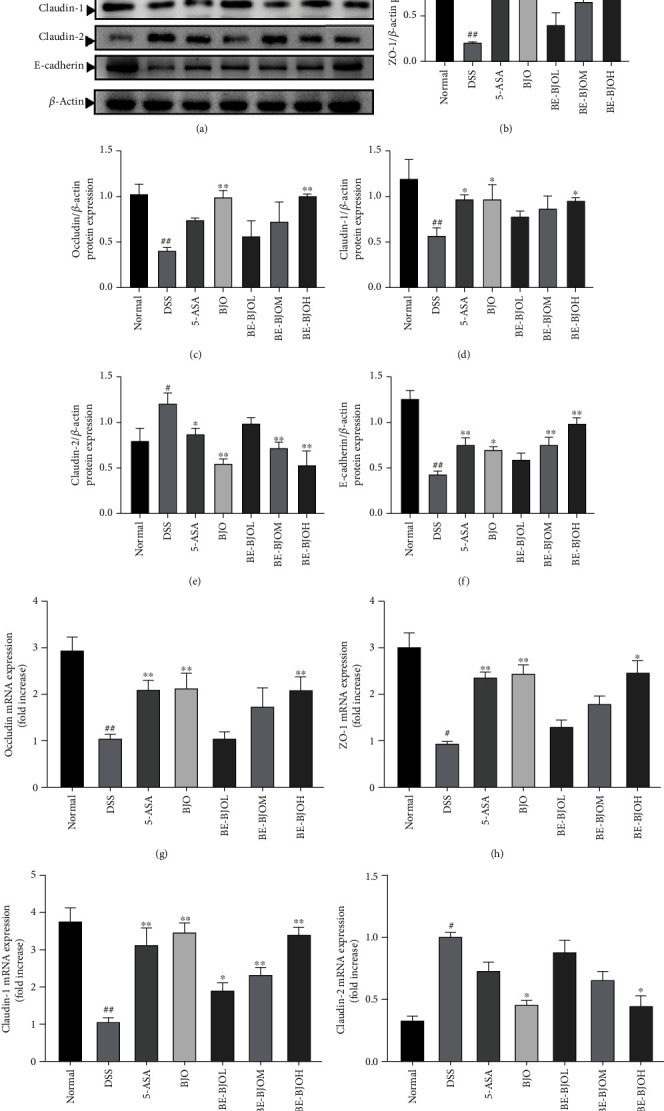
BE-BJO restored intestinal barrier function in DSS-induced colitis in mice. Representative protein expression of ZO-1, occludin, claudin-1, claudin-2, and E-cadherin (a) in colon tissue. The bar graph of the relative intensities of ZO-1 (b), occludin (c), claudin-1 (d), claudin-2 (e), and E-cadherin (f) Western Blotting bands. The mRNA expression of occludin (g), ZO-1 (h), and claudin-1 (i) and claudin-2 (j) in colon tissue. All values are presented as the mean ± SEM. ^##^*P* < 0.01 and ^#^*P* < 0.05 versus normal group; ^∗^*P* < 0.05, ^∗∗^*P* < 0.01, and ^∗∗∗^*P* < 0.001 versus DSS group.

Since UC is characterized by damage of intestinal mucosal barrier function, therapeutics that could enhance intestinal mucosal healing is a major goal of UC therapy [20, 25]. The intestinal epithelial barrier function depends on the integrity of the mucus layer, which is determined by the expression and assembly of tight junction proteins [26, 27]. The tight junction complex is constituted by transmembrane proteins, like occludin and the claudin family, and by linker proteins, like zonula occludens-1 (ZO-1), that affiliate with the actin cytoskeleton [28]. To examine the effect of BE-BJO on intestinal mucosal healing, the expressions of tight junction proteins were tested. As shown in Figure 5, treatment of DSS for 7 days resulted in a remarkable decrease in the protein expressions of ZO-1, occludin, claudin-1, and E-cadherin but augmented the expression of claudin-2 as compared to the control group. Treatment of BE-BJO reversed the effect of DSS on expressions of these tight junction proteins in a dose-dependent manner. A high dose of BE-BJO shares a parallel effect with BJO and the positive drug 5-ASA. These observations imply that BE-BJO could enhance intestinal epithelial barrier function by altering the expression levels of tight junction proteins.

### 3.5. BE-BJO Suppressed NF-*κ*B Activation in DSS-Induced Colitis

**Figure 6:**
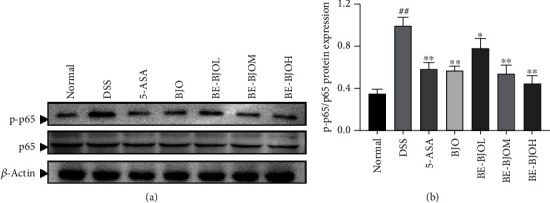
BE-BJO inhibited the activation of the NF-*κ*B signaling pathway. Representative Western Blot images of p-65 and p-p65 (a). The relative protein expression of p-p65 (b) in colon tissue was detected by Western Blot. All values are presented as the mean ± SEM. ^##^*P* < 0.01 versus normal group; ^∗^*P* < 0.05, ^∗∗^*P* < 0.01, and ^∗∗∗^*P* < 0.001 versus DSS group.

The phosphorylation of p65 (p-p65) is a hallmark of NF-*κ*B activation and leads to colon inflammation during the pathogenesis of UC [29, 30]. Thus, the levels of p-p65 and total p65 in the colon tissues of mice were determined by Western Blot. The results demonstrated that DSS treatment significantly upregulated the expression of p-p65, without altering the expression of total p65 (Figure 6). Treatment with 5-ASA, BJO, and BE-BJO (152.5, 305, and 610 mg/kg/day) significantly reduced the level of p-p65 and the ratio of p-p65/p65 (Figure 6). Thus, the results indicate that the BE-BJO could repress NF-*κ*B activation through inhibiting the phosphorylation of p65.

### 3.6. BE-BJO Inhibited the Activation of the RhoA/ROCK Signaling Pathway in DSS-Induced Colitis

**Figure 7:**
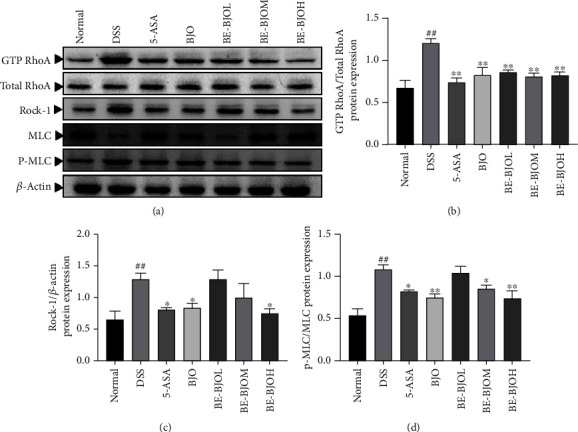
BE-BJO inhibited the activation of the RhoA/ROCK signaling pathway in DSS-induced UC in mice. Representative Western Blot images of GTP RhoA, total RhoA, ROCK-1, p-MLC, and MLC (a). The relative protein expressions of GTP RhoA (b), ROCK-1 (c), and p-MLC (d) in colon tissue were detected by Western Blot. All values are presented as the mean ± SEM. ^##^*P* < 0.01 versus normal group; ^∗^*P* < 0.05, ^∗∗^*P* < 0.01, and ^∗∗∗^*P* < 0.001 versus DSS group.

RhoA/ROCK is closely associated with the regulation of adhesion, migration, and proliferation of intestinal crypt cells, thus playing a pivotal role in the development of UC [7]. We therefore investigated the effect of BE-BJO on the RhoA/ROCK signaling pathway. Firstly, the activation of RhoA was determined by RhoA activity assay. As shown in Figures 7(a) and 7(b), DSS facilitated the activation of RhoA, as implied by the increased ratio of GTP-RhoA/total RhoA. BE-BJO, BJO, and 5-ASA significantly repressed RhoA activation. Secondly, the protein expression of ROCK-1 was augmented by DSS but was recovered to the normal level in the presence of BE-BJO (610 mg/kg/day), BJO, or 5-ASA (Figures 7(a) and 7(c)). Thirdly, the phosphorylation level of myosin light chain (MLC), which serves as a substrate of ROCK-1, was determined. BE-BJO (305, 610 mg/kg/day), BJO, and 5-ASA significantly reversed DSS-induced phosphorylation of MLC (Figures 7(a) and 7(d)). Therefore, these data confirm that BE-BJO could suppress the RhoA/ROCK signaling pathway.

## 4. Discussion

BJO is well-accepted to have antidiarrheal properties and has been reported to have an anti-inflammatory effect in UC disease models, thus suggesting therapeutic potential in the treatment of UC [12]. Besides quassinoids including brusatol and bruceine D, BJO also contains oleic acid, linoleic acid, stearic acid, palmitic acid, arachidonic acid, and other unsaturated fatty acids [31]. In order to elucidate whether or not these components contribute to the anti-UC properties of BJO, the present study compared the effects of BE-BJO and BF-BJO in the DSS-induced UC model. According to our observations, BE-BJO alleviated DSS-induced loss of body weight, an increase of DAI, and a shortening of colon, whereas BF-BJO did not have these protective effects. The effect of BE-BJO is equivalent to that of BJO. It thus suggests that quassinoids in particular brusatol, but not oleic acid or other unsaturated fatty acids, serve as the active components for the anti-UC properties of BJO.

UC is a progressive alimentary system disorder that is characterized by dysregulated immune response, chronic inflammation, unbalanced gut microbiota (dysbiosis), and defective mucosal barrier function [32, 33]. The present study further explored the effects and mechanisms of BE-BJO on colon inflammation and intestinal epithelial barrier function.

In line with our previous observations [11, 12], the present results showed that treatment of BE-BJO suppressed the production and release of proinflammatory cytokines including TNF-*α*, IFN-*γ*, IL-6, and IL-1*β* in the colon tissue. TNF-*α* is a proinflammatory molecule that mediates multiple physiological and pathological processes of UC. Excess production of TNF-*α* leads to the secretion of chemokines by colonic epithelial cells and damage of epithelial barrier [34, 35]. IL-1*β* emerges as a requisite role in the development of inflammatory reaction, and the increase of IL-1*β* level can cause autoimmune process and then impairs colon tissue [36, 37]. IL-6 is an immunomodulatory cytokine that participates in the progression of UC [38, 39]. IL-6 and IL-1*β* are team players in the development of UC. INF-*γ* is an activator of phagocytes and neutrophil with immunomodulatory activity [40]. Thus, inhibiting the release of these proinflammatory mediators contributes to the anti-inflammatory properties of BJO.

NF-*κ*B is pivotal in the inflammatory cascades during the pathogenesis of colitis [41]. The p65 subunit is the major functional subunit in NF-*κ*B. In response to stimulation, p65 undergoes phosphorylation and is transferred to the nucleus, where it binds to promoters of target genes to enhance the inflammatory response [6, 42]. Our results demonstrated that BE-BJO dose-dependently reversed DSS-induced p65 phosphorylation, thus suggesting that BE-BJO could repress NF-*κ*B activation.

Moreover, the present study indicates that the anti-UC effect of BE-BJO is attributed to improvement of intestinal stability and structural integrity by enhancing tight junctions. The primary structure of intestinal barrier is the apical junctional complex, which consists of the tight junctions and the subjacent adherent junctions that contribute to apical-basal cell polarity maintenance and to cell signaling events [43]. The tight junction complex is constituted by transmembrane proteins such as occludin and the claudin family and by linker proteins like ZO-1 [28]. These proteins regulate the permeability and integrity of intestinal mucosal barrier [44, 45]. In this study, BE-BJO rebounded the decreased expressions of ZO-1, occludin, claudin-1, and E-cadherin induced by DSS but augmented claudin-2 expression, thus indicating that BE-BJO could regulate the expressions of colonic epithelial barrier-associated proteins, thereby repairing the damaged colonic mucosa during UC. Taken into considerations that therapeutics targeting enhancing intestinal mucosal healing is a major goal of UC therapy [25, 46], the present findings provide important evidence for the therapeutic potential of BJO in the treatment of UC.

To elucidate the mechanisms underlying the effect of BJO on intestinal epithelial barrier function, we tested the possible involvement of RhoA/ROCK signaling. ROCK and myosin light chain kinases (MLCK) are involved in the regulation of tight junctions during the intestinal inflammatory response [47, 48]. Activated RhoA/ROCK signals deactivate myosin phosphatase (MLCP), leading to failure of dephosphorylation of MLC, resulting in increased levels of intracellular phosphorylation and increased actin-myosin cross-linking, finally promoting the polymerization of the actin microfilaments [49, 50]. In addition, RhoA/ROCK elevates MLC phosphorylation levels. The present study indicates that BE-BJO inhibits the RhoA/ROCK signaling pathway. This conclusion is supported by the following observations: (1) BE-BJO repressed DSS-induced activation of RhoA; (2) BE-BJO treatment downregulated ROCK expression; and (3) BE-BJO prevented phosphorylation of MCL in DSS-treated mice. Since the RhoA/ROCK signaling pathway is associated with colon inflammation and regulation of intestinal epithelial barrier function, it is probably that BE-BJO ameliorated UC by repressing RhoA/ROCK. Intriguingly, there is a crosstalk between NF-*κ*B and RhoA/ROCK which work together to accelerate the progression of UC [33, 51], the inhibitory effect of BE-BJO on both signaling pathways might help to explain the remarkable anti-UC effect of BE-BJO.

## 5. Conclusion

In conclusion, the present study has shown that BJO enriched with brusatol could ameliorate DSS-induced UC by preventing colon inflammation and enhancing intestinal epithelial barrier function. Mechanistically, these anti-UC effects of BE-BJO are probably associated with repression of NF-*κ*B and RhoA/ROCK signaling pathways. These findings confirm that brusatol is the active compound from BJO and suggest the therapeutic potential of brusatol and BE-BJO in the treatment of UC.

## Figures and Tables

**Table 1:** The criteria of DAI.

Body weight loss	Stool consistency	Occult blood or bloody stool	Score
No change	Normal	Negative	0
1-5%	Loose stool	Negative	1
5-10%	Loose stool	Occult blood positive	2
10-20%	Diarrhea	Occult blood positive	3
≥20%	Diarrhea	Gross hematochezia	4

**Table 2:** The criteria of a histological score.

Categories	Feature	Score
Architectural changes	No change	0
	Mild abnormality in mucosa	1
	Moderate diffuse or multifocal abnormalities in submucosa	2
	Severe diffuse or multifocal abnormalities	3
	Peritonitis	4

Inflammatory infiltrate	No change	0
	Mild but unequivocal increase	1
	Moderate change	2
	Marked change	3
	Severe increase	4

Lamina propria leukocytes	No changes	0
	Mild increase	1
	Moderate increase	2
	Marked increase	3
	Severe increase	4

Intraepithelial neutrophils	No changes	0
	25%	1
	50%	2
	75%	3
	100%	4

Erosion or ulceration	No change	0
	Erosion focally stripped	1
	Marked erosion	2
	Severe erosion	3
	Ulceration or granulation tissue	4

Crypt destruction	No change	0
	Blunted crypts	1
	Marked attenuation	2
	Crypt necrosis	3
	No architecture	4

**Table 3:** Primer sequences for qRT-PCR.

Gene		Primer (5′ to 3′)
ZO-1	Forward	CACCTCGCACGCATCACAGC
	Reverse	GGCGGCAATGGTGGTCCTTC
Occludin	Forward	TTGGCTACGGAGGTGGCTATGG
	Reverse	CCTTTGGCTGCTCTTGGGTCTG
Claudin-1	Forward	TGGCTTCTCTGGGATGGATCGG
	Reverse	CCTGAGCGGTCACGATGTTGTC
Claudin-2	Forward	CCGTGTTCTGCCAGGATTCTCG
	Reverse	AGCCCAGGATGCCACCAAGG
GAPDH	Forward	GGTTGTCTCCTGCGACTTCA
	Reverse	TGGTCCAGGGTTTCTTACTCC

## Data Availability

The data that support the findings of this study are available from the corresponding author upon request.

## Abbreviations

5-ASA: 5-Aminosalicylic acid
BE-BJO: Brusatol-enriched BJO
BF-BJO: Brusatol-free BJO
BJO: Brucea javanica oil
DAI: Disease activity index
DSS: Dextran sulfate sodium
Elisa: Enzyme-linked immunosorbent assay
HE: Hematoxylin-eosin
HPLC: High-performance liquid chromatography
IBD: Inflammatory bowel disease
IFN: Interferon
IL-1*β*: Interleukin-1*β*
IL-6: Interleukin-6
MLC: Myosin light chain
MLCK: Myosin light chain kinase
NF-*κ*B: Nuclear factor kappa-B
RT-PCR: Reverse transcription-polymerase chain reaction
ROCK: Rho-associated kinase
TGF-*β*: Transforming growth factor-*β*
TNF-*α*: Tumor necrosis factor-*α*
TJ: Tight junction
UC: Ulcerative colitis
ZO: Zonula occluden.

## References

[1] Szandruk M., Merwid-Lad A., Szelag A. (2018). The impact of mangiferin from Belamcanda chinensis on experimental colitis in rats. *Inflammopharmacology*.

[2] Zong S.-y., Pu Y.-q., Xu B.-l., Zhang T., Wang B. (2017). Study on the physicochemical properties and anti-inflammatory effects of paeonol in rats with TNBS-induced ulcerative colitis. *International Immunopharmacology*.

[3] Zhang D.-K., Yu J.-J., Li Y.-M. (2012). A *Picrorhiza kurroa* derivative, picroliv, attenuates the development of dextran-sulfate-sodium-induced colitis in mice. *Mediators of Inflammation*.

[4] Seril D. N., Liao J., Yang G.-Y. (2007). Colorectal carcinoma development in inducible nitric oxide synthase-deficient mice with dextran sulfate sodium-induced ulcerative colitis. *Molecular Carcinogenesis*.

[5] Yu X., Yang G., Jiang H. (2017). Patchouli oil ameliorates acute colitis: a targeted metabolite analysis of 2, 4, 6-trinitrobenzenesulfonic acid-induced rats. *Experimental and Therapeutic Medicine*.

[6] Christian J., Vier J., Paschen S. A., Häcker G. (2010). Cleavage of the NF-*κ*B Family Protein p65/RelA by the Chlamydial Protease-like Activity Factor (CPAF) Impairs Proinflammatory Signaling in Cells Infected with Chlamydiae. *Journal of Biological Chemistry*.

[7] Citalan-Madrid A. F., Vargas-Robles H., Garcia-Ponce A. (2017). Cortactin deficiency causes increased RhoA/ROCK1-dependent actomyosin contractility, intestinal epithelial barrier dysfunction, and disproportionately severe DSS-induced colitis. *Mucosal Immunology*.

[8] Yang J., Li S., Xie C. (2013). Anti-inflammatory activity of ethyl acetate fraction of the seeds of *Brucea Javanica*. *Journal of Ethnopharmacology*.

[9] Qiu Z. H., Zhang W. W., Zhang H. H., Jiao G. H. (2019). Brucea javanicaoil emulsion improves the effect of radiotherapy on esophageal cancer cells by inhibiting cyclin D1-CDK4/6 axis. *World Journal of Gastroenterology*.

[10] Shao A., Chen G., Jiang N. (2013). Development and evaluation of self-microemulsifying liquid and granule formulations of Brucea javanica oil. *Archives of Pharmacal Research*.

[11] Huang Y. F., Zhou J. T., Qu C. (2017). Anti-inflammatory effects of *Brucea javanica* oil emulsion by suppressing NF-*κ*B activation on dextran sulfate sodium-induced ulcerative colitis in mice. *Journal of Ethnopharmacology*.

[12] Huang Y. F., Li Q. P., Dou Y. X. (2019). Therapeutic effect of *Brucea javanica* oil emulsion on experimental Crohn 's disease in rats: Involvement of TLR4/ NF-*κ*B signaling pathway. *Biomedicine & Pharmacotherapy*.

[13] Chumkaew P., Srisawat T. (2017). Antimalarial and cytotoxic quassinoids from the roots ofBrucea javanica. *Journal of Asian Natural Products Research*.

[14] Zhan Y., Tan T., Qian K., Yang S., Feng Y., Wen Q. (2020). Quassinoids from seeds ofBrucea Javanicaand their anticomplement activities. *Natural Product Research*.

[15] Dou Y.-X., Zhou J.-T., Wang T.-T. (2018). Self-nanoemulsifying drug delivery system of bruceine D: a new approach for anti-ulcerative colitis. *International Journal of Nanomedicine*.

[16] Zhou J., Wang T., Dou Y. (2018). Brusatol ameliorates 2, 4, 6-trinitrobenzenesulfonic acid-induced experimental colitis in rats: Involvement of NF-*κ*B pathway and NLRP3 inflammasome. *International Immunopharmacology*.

[17] Wang T., Dou Y., Lin G. (2020). The anti-hepatocellular carcinoma effect of Brucea javanica oil in ascitic tumor-bearing mice: the detection of brusatol and its role. *Biomedicine & Pharmacotherapy*.

[18] Zou Y. T., Ma L. L., Zhao Y., Zhang S. C., Zhou C. H., Cai Y. (2018). Inhibition of Rho kinase protects against colitis in mice by attenuating intestinal epithelial barrier dysfunction via MLC and the NF-*κ*B pathway. *International Journal of Molecular Medicine*.

[19] Kim J. J., Shajib M. S., Manocha M. M., Khan W. I. (2012). Investigating intestinal inflammation in DSS-induced model of IBD. *Journal of Visualized Experiments*.

[20] Nguyen H. T. T., Dalmasso G., Müller S., Carrière J., Seibold F., Darfeuille–Michaud A. (2014). Crohn's Disease-Associated Adherent Invasive *Escherichia coli* Modulate Levels of microRNAs in Intestinal Epithelial Cells to Reduce Autophagy. *Gastroenterology*.

[21] Andrade M. E. R., Barros P. A. V., Menta P. L. . R. (2019). Arginine supplementation reduces colonic injury, inflammation and oxidative stress of DSS-induced colitis in mice. *Journal of Functional Foods*.

[22] Camuesco D., Gálvez J., Nieto A. (2005). Dietary olive oil supplemented with fish oil, rich in EPA and DHA (n-3) polyunsaturated fatty acids, attenuates colonic inflammation in rats with DSS-induced colitis. *The Journal of Nutrition*.

[23] Larrosa M., Yañéz-Gascón M. J., Selma M. V. (2009). Effect of a low dose of dietary resveratrol on colon microbiota, inflammation and tissue damage in a DSS-induced colitis rat model,. *Journal of Agricultural and Food Chemistry*.

[24] Wang Y., Jiang X., Zhu J. (2016). IL-21/IL-21R signaling suppresses intestinal inflammation induced by DSS through regulation of Th responses in lamina propria in mice. *Scientific Reports*.

[25] Atreya R., Neurath M. F. (2017). Current and future targets for mucosal healing in inflammatory bowel disease. *Visceral Medicine*.

[26] Zeisel M. B., Dhawan P., Baumert T. F. (2019). Tight junction proteins in gastrointestinal and liver disease. *Gut*.

[27] Putt K. K., Pei R., White H. M., Bolling B. W. (2017). Yogurt inhibits intestinal barrier dysfunction in Caco-2 cells by increasing tight junctions. *Food & Function*.

[28] Mennigen R., Nolte K., Rijcken E. (2009). Probiotic mixture VSL#3 protects the epithelial barrier by maintaining tight junction protein expression and preventing apoptosis in a murine model of colitis. *American Journal of Physiology-Gastrointestinal and Liver Physiology*.

[29] Murano M., Maemura K., Hirata I. (2000). Therapeutic effect of intracolonically administered nuclear factor kappa B (p65) antisense oligonucleotide on mouse dextran sulphate sodium (DSS)-induced colitis. *Clinical & Experimental Immunology*.

[30] Hegazy S. K., El-Bedewy M. M. (2010). Effect of probiotics on pro-inflammatory cytokines and NF-*κ*B activation in ulcerative colitis. *World Journal of Gastroenterology*.

[31] Ma S., Chen F., Ye X. (2013). Intravenous microemulsion of docetaxel containing an anti-tumor synergistic ingredient (Brucea javanica oil): formulation and pharmacokinetics. *International Journal of Nanomedicine*.

[32] Ungaro R., Mehandru S., Allen P. B., Peyrin-Biroulet L., Colombel J.-F. (2017). Ulcerative colitis. *The Lancet*.

[33] Ma J., Yin G., Lu Z. (2018). Casticin prevents DSS induced ulcerative colitis in mice through inhibitions of NF-*κ*B pathway and ROS signaling. *Phytotherapy Research*.

[34] Akdis M., Aab A., Altunbulakli C. (2016). Interleukins (from IL-1 to IL-38), interferons, transforming growth factor *β*, and TNF-*α*: Receptors, functions, and roles in diseases. *Journal of Allergy and Clinical Immunology*.

[35] Ungar B., Levy I., Yavne Y. (2016). Optimizing Anti-TNF-*α* Therapy: Serum Levels of Infliximab and Adalimumab Are Associated With Mucosal Healing in Patients With Inflammatory Bowel Diseases. *Clinical Gastroenterology and Hepatology*.

[36] Karmakar M., Katsnelson M., Malak H. A. (2015). Neutrophil IL-1*β* processing induced by pneumolysin is mediated by the NLRP3/ASC inflammasome and caspase-1 activation and is dependent on K^+^ Efflux. *The Journal of Immunology*.

[37] Dror E., Dalmas E., Meier D. T. (2017). Postprandial macrophage-derived IL-1*β* stimulates insulin, and both synergistically promote glucose disposal and inflammation. *Nature Immunology*.

[38] Gupta R. A., Motiwala M. N., Mahajan U. N., Sabre S. G. (2018). Protective effect of *Sesbania grandiflora* on acetic acid induced ulcerative colitis in mice by inhibition of TNF- *α* and IL-6. *Journal of Ethnopharmacology*.

[39] Grivennikov S., Karin E., Terzic J. (2009). IL-6 and Stat3 are required for survival of intestinal epithelial cells and development of colitis-associated cancer. *Cancer Cell*.

[40] Tenger C., Sundborger A., Jawien J., Zhou X. H. (2005). IL-18 accelerates atherosclerosis accompanied by elevation of IFN-*γ* and CXCL16 expression independently of T cells. *Arteriosclerosis Thrombosis and Vascular Biology*.

[41] Zhang X., Du Q., Yang Y. (2017). The protective effect of Luteolin on myocardial ischemia/reperfusion (I/R) injury through TLR4/NF-*κ*B/NLRP3 inflammasome pathway. *Biomedicine & Pharmacotherapy*.

[42] Sandur S. K., Ichikawa H., Sethi G., Ahn K. S., Aggarwal B. B. (2006). Plumbagin (5-Hydroxy-2-methyl-1,4-naphthoquinone) Suppresses NF-*κ*B Activation and NF-*κ*B-regulated Gene Products Through Modulation of p65 and I*κ*B*α* Kinase Activation, Leading to Potentiation of Apoptosis Induced by Cytokine and Chemotherapeutic Agents. *Journal of Biological Chemistry*.

[43] Gehren A. S., Rocha M. R., de Souza W. F., Morgado-Diaz J. A. (2015). Alterations of the apical junctional complex and actin cytoskeleton and their role in colorectal cancer progression. *Tissue Barriers*.

[44] Coopman P., Djiane A. (2016). Adherens junction and E-cadherin complex regulation by epithelial polarity. *Cellular and Molecular Life Sciences*.

[45] Rübsam M., Mertz A. F., Kubo A. (2017). E-cadherin integrates mechanotransduction and EGFR signaling to control junctional tissue polarization and tight junction positioning. *Nature Communications*.

[46] Brown S. J., Mayer L. (2007). The immune response in inflammatory bowel disease. *The American Journal of Gastroenterology*.

[47] Walsh S. V., Hopkins A. M., Chen J., Narumiya S., Parkos C. A., Nusrat A. (2001). Rho kinase regulates tight junction function and is necessary for tight junction assembly in polarized intestinal epithelia. *Gastroenterology*.

[48] Cetin S., Ford H. R., Sysko L. R. (2004). Endotoxin Inhibits Intestinal Epithelial Restitution through Activation of Rho-GTPase and Increased Focal Adhesions. *Journal of Biological Chemistry*.

[49] Tang S.-t., Su H., Zhang Q. (2016). Melatonin attenuates aortic endothelial permeability and arteriosclerosis in streptozotocin-induced diabetic rats: possible role of MLCK- and MLCP-dependent MLC phosphorylation. *Journal of Cardiovascular Pharmacology and Therapeutics*.

[50] Kang H., Liu J., Sun A., Liu X., Fan Y., Deng X. (2017). Vascular smooth muscle cell glycocalyx mediates shear stress-induced contractile responses via a Rho kinase (ROCK)-myosin light chain phosphatase (MLCP) pathway. *Scientific Reports*.

[51] Elamin E., Masclee A., Dekker J., Jonkers D. (2014). Ethanol disrupts intestinal epithelial tight junction integrity through intracellular calcium-mediated Rho/ROCK activation. *American Journal of Physiology-Gastrointestinal and Liver Physiology*.

